# Genome-scale identification of cell-wall related genes in *Arabidopsis* based on co-expression network analysis

**DOI:** 10.1186/1471-2229-12-138

**Published:** 2012-08-09

**Authors:** Shan Wang, Yanbin Yin, Qin Ma, Xiaojia Tang, Dongyun Hao, Ying Xu

**Affiliations:** 1 Computational Systems Biology Laboratory, Department of Biochemistry and Molecular Biology, and Institute of Bioinformatics, Athens, GA, USA; 2 BESC BioEerngy Science Center, University of Georgia, Athens, GA, USA; 3 Key Lab for Molecular Enzymology and Engineering of the Ministry of Education, Jilin University, Changchun, China; 4 Biotechnology Research Centre, Jilin Academy of Agricultural Sciences (JAAS), Changchun, China; 5 College of Computer Science and Technology, Jilin University, Changchun, China

**Keywords:** Plant cell wall, *Arabidopsis*, Co-expression network analysis, Bi-clustering, *Cis* regulatory motifs

## Abstract

**Background:**

Identification of the novel genes relevant to plant cell-wall (PCW) synthesis represents a highly important and challenging problem. Although substantial efforts have been invested into studying this problem, the vast majority of the PCW related genes remain unknown.

**Results:**

Here we present a computational study focused on identification of the novel PCW genes in *Arabidopsis* based on the co-expression analyses of transcriptomic data collected under 351 conditions, using a bi-clustering technique. Our analysis identified 217 highly co-expressed gene clusters (modules) under some experimental conditions, each containing at least one gene annotated as PCW related according to the Purdue Cell Wall Gene Families database. These co-expression modules cover 349 known/annotated PCW genes and 2,438 new candidates. For each candidate gene, we annotated the specific PCW synthesis stages in which it is involved and predicted the detailed function. In addition, for the co-expressed genes in each module, we predicted and analyzed their *cis* regulatory motifs in the promoters using our motif discovery pipeline, providing strong evidence that the genes in each co-expression module are transcriptionally co-regulated. From the all co-expression modules, we infer that 108 modules are related to four major PCW synthesis components, using three complementary methods.

**Conclusions:**

We believe our approach and data presented here will be useful for further identification and characterization of PCW genes. All the predicted PCW genes, co-expression modules, motifs and their annotations are available at a web-based database: http://csbl.bmb.uga.edu/publications/materials/shanwang/CWRPdb/index.html.

## Background

Plant cell walls (PCWs) are mainly composed of polysaccharides and lignins, forming the major component of plant biomass. Knowing which genes are involved in the formation and remodeling of PCWs is of great importance as they play many critical roles during plant growth, including regulation of cell differentiation, intercellular adhesion and communication, control of water movement, and defense against invasions by pests and pathogens [1-4], not to mention that it is the focal point of cellulosic biofuel studies. It is estimated that genes involved in the PCW synthesis, remodeling and turnover may account for about 15% of all ~26,500 protein-encoding genes in *Arabidopsis* genome [4,5], i.e., ~4,000 genes. As of today only ~1,000 *Arabidopsis* genes have been characterized or predicted to be PCW related according to the Purdue Cell Wall Gene Families database (the Purdue database hereafter) [6]. Hence, the vast majority of the PCW related genes in *Arabidopsis* genes are yet to be identified.

Experimental elucidation of PCW related genes have been mainly done through forward genetic screening [7,8], which is time consuming and expensive. The rapid accumulation of genome-scale gene-expression data allows computational prediction of PCW related genes through co-expression analyses. The basic idea is that genes deemed to be co-expressed under multiple conditions tend to be functionally related [9-11]; hence genes that are co-expressed with known PCW genes may also be PCW related. A number of studies have been carried out for inference of PCW related genes using this or similar ideas. For example, Brown *et al.* and Persson *et al.* published the first two studies on prediction of new PCW related genes through microarray data analyses [12,13], in which cellulose synthesis (CESA) genes, CESA4, CESA7, and CESA8 were used as the ‘seeds’ to identify additional genes with the similar expression patterns*.* A high percentage of the genes predicted to be PCW related in the two studies were later experimentally verified to be indeed involved in PCW biosynthesis [14-16], which demonstrated the power of co-expression analyses in identifying potential PCW genes, providing good candidates for further experimental validation.

We present here a study on prediction of novel PCW related genes in *Arabidopsis* at a genome scale based on the published gene-expression data collected under 351 conditions [17]. An unique feature of our study, compared to the previous similar studies, is that we aim to find genes co-expressed with the known PCW related genes under multiple but not necessarily all conditions. This makes our strategy substantially more sensitive and specific in detection of the PCW related genes compared to the published studies [12,13]. But this also raised a very challenging technical problem: how to determine which subsets of the 351 conditions should be considered? Clearly it is unrealistic to exhaustively go through all 2^351^ subsets with at least certain size to search for such co-expressed genes.

To overcome this issue, we have applied a new and generalized clustering technique, called *bi-clustering*[18-20], to search for gene groups co-expressed under some (to-be-identified) of the 351 conditions. We specifically employed QUBIC, a bi-clustering algorithm that we recently developed for solving this type of generalized clustering problem [21].

We have implemented a computational pipeline based on QUBIC to perform bi-clustering analyses of the 351 transcriptomic datasets using the known/annotated PCW related genes (the known PCW genes hereafter) as seeds to generate co-expressed gene modules in *Arabidopsis*. The predicted co-expressed gene modules were then computationally validated to be transcriptionally co-regulated through identification of conserved *cis* regulatory motifs in the promoters of genes in the same module. Using this approach we identified 2,438 candidate genes that are co-expressed with 349 known PCW genes under some conditions with high statistical significance. Functional analyses on the candidate genes revealed more detailed functional roles of these genes in PCW synthesis and remodeling. We have carried out detailed functional analyses of the co-expression modules containing the genes related to four major PCW synthesis components, which are likely to encode biological pathways with similar functions but are expressed under distinct conditions. We believe that our overall analysis procedure will be useful for gene expression data analysis in elucidation of other biological pathways in plants in general.

## Results and discussion

### Computational pipeline for inference of co-expressed PCW genes

**Figure 1:**
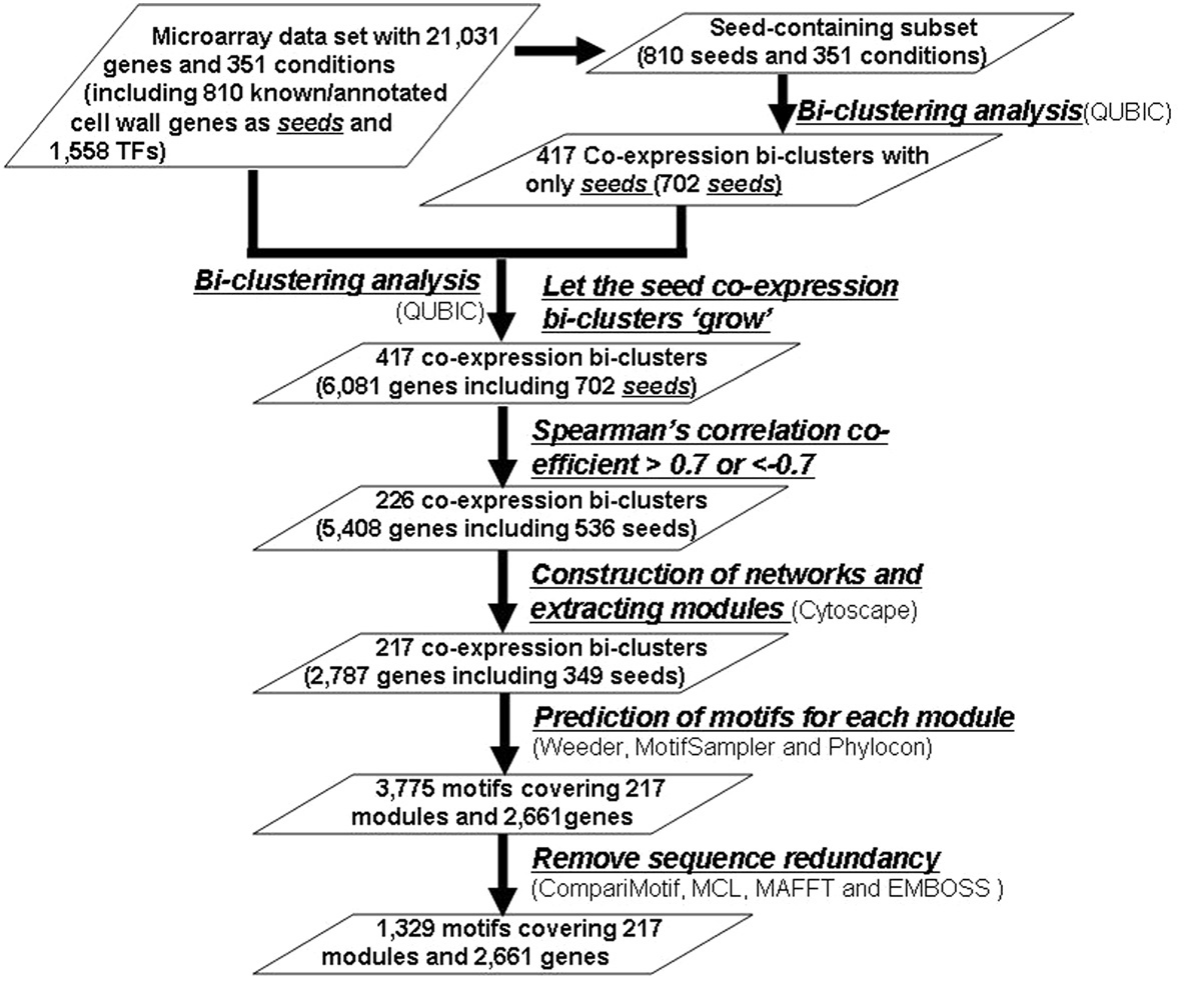
A flowchart of the computational analysis pipeline.

To identify genes co-expressed with the known PCW genes, we developed a computational pipeline (Figure 1). The pipeline consists of the following steps: (1) identification of co-expressed genes among the known PCW genes using the bi-clustering program QUBIC; (2) expansion of the bi-clusters to include additional genes under the same conditions which are previously unknown to be PCW related; (3) reconstruction of a co-expression gene network containing both known PCW genes and newly recruited genes based on each expanded bi-cluster; (4) extraction of sub-networks, named *co-expression module,* within each network; and (5) prediction, integration and annotation of conserved motifs in the promoter regions of co-expressed genes within each module.

### Co-expression modules of PCW genes

Among the 810 known PCW genes, a total of 217 co-expression modules are identified, which cover 349 known PCW genes (Additional file 1: Table S1). These genes cover five of the six key stages related to PCW syntheses in the Purdue database, namely *substrate generation*; *polysaccharide synthases and glycosyl transferases*; *assembly, architecture and growth*; *differentiation and secondary wall formation;* and *signaling and response* without any genes involved in *secretion and targeting*, which might be due to the fact that only a handful of genes are known to be involved in this stage. Another reason could be its low gene-expression correlation with other stages, since its machinery is dynamically coupled with cytoskeleton [22].

We have assessed the quality of the predicted modules that contain the known CESA genes responsible for secondary wall cellulose, namely CESA4, CESA7 and CESA8, which have been widely studied and well annotated [12,13,23]. We use the assessment results on these genes as an indicator of the overall quality of the 217 predicted modules as quality assessment of all these modules is not doable at this point due to the lack of the ground truth information for the most of them.

**Figure 2:**
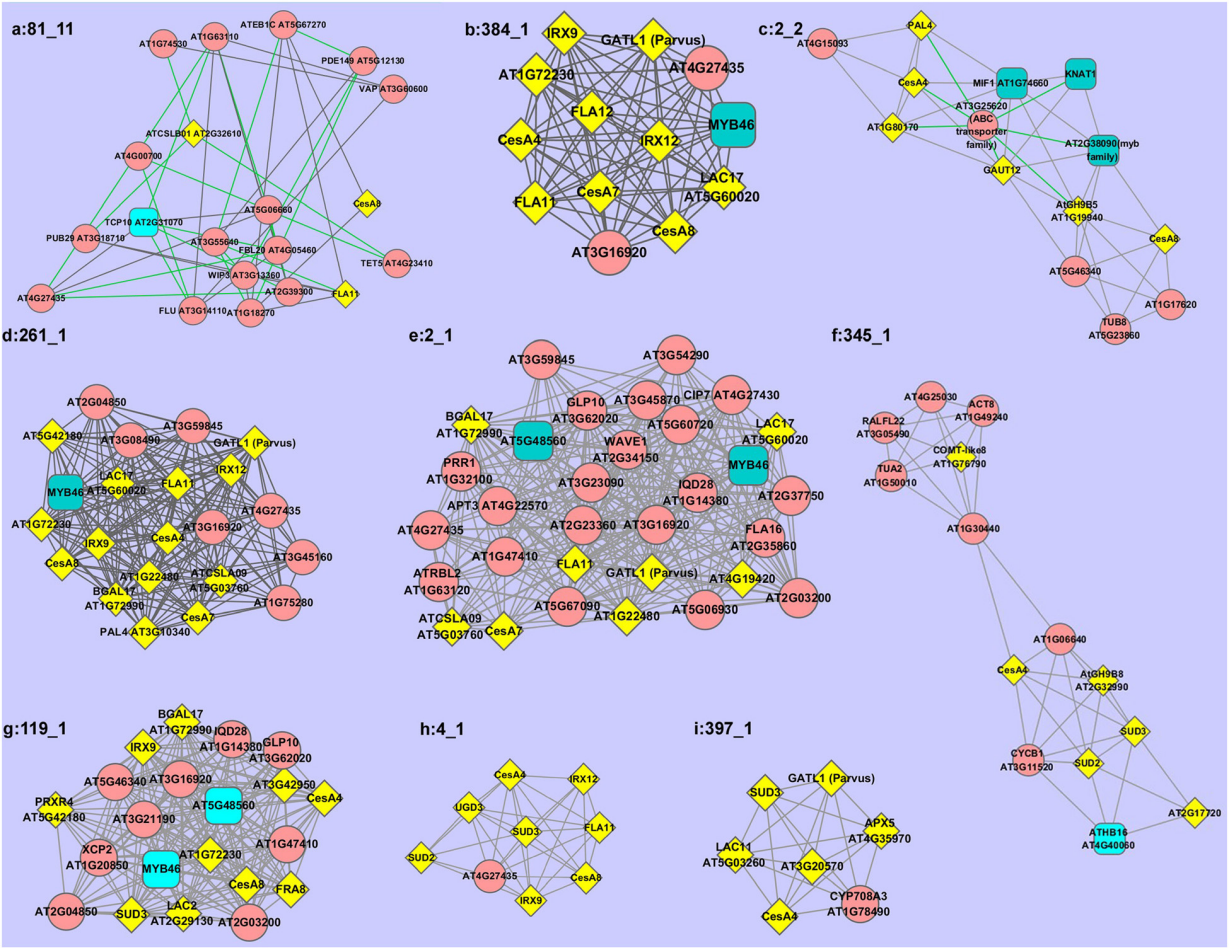
**Nine co-expression modules containing CESA genes of CESA4, CESA7, and CESA8.** Each square represents a TF; each yellow diamond represents a known PCW gene, and a red circle represents a novel PCW gene. Wherever possible, gene names are used instead of TAIR’s AT numbers. CESA4: AT5G44030, CESA7: AT5G17420, CESA8: AT4G18780, IRX9: AT2G37090, GAUT12/IRX8: AT5G54690, IRX12: AT2G38080, FRA8: AT2G28110, GATL1/Parvus: AT1G19300, SUD2: AT5G59290, SUD3: AT2G28760, PAL4: AT3G10340, UGD3: AT5G15490, COMT-like8: AT1G76790.

We noted that 9 modules each contain at least one of three CESA genes (Figure 2). Each of these modules also contains many of the genes previously reported or predicted to be co-expressed with CESA genes [12,13,17,24], such as the GT8 family gene GATL1/Parvus (in modules *261_1, 384_1, 4_1, 397_1*) and GAUT12/IRX8 (in module *2_2*), the GT47 family gene FRA8 (in module *119_1*), the GT43 family gene IRX9 (in modules *119_1, 261_1, 384_1, 4_1*), and lignin synthesis related gene IRX12 (in modules *261_1, 384_1, 4_1*) [13]. Besides, the transcription factors (TFs) of MYB46 (in modules *2_1, 119_1, 261_1, 384_1*), known as the master switch for secondary cell wall synthesis [25], is recently reported to be co-expressed with CESA genes [23]. 

### Functional distribution of the candidate PCW genes

Our bi-clustering analysis predicted 2,438 candidate PCW genes, which are co-expressed with 349 known PCW genes in 217 modules (Additional file 1: Tables S2 and S3). 190 of these modules (88%) contain no more than 40 genes (Additional file 2: Figure S1). 74 modules out of the 217 ones contain seed genes from only one PCW synthesis stage. 33 of those have over 10% of their genes being seed genes. A total of 201 novel PCW genes in these 33 modules were predicted to be involved in a specific synthesis stage (Additional file 1: Table S4).

To assess the experimental conditions associated with each module, we extracted the tissue/organ information associated with the co-expression conditions in each module (Additional file 1: Tables S2, S3). These modules covered 317 out of the total of 351 conditions, related to 62 different tissue/organs. We performed Plant Ontology (PO) anatomy enrichment analyses [26] on both the seed and the total genes in each module, and retained the top five PO enriched tissues/organs (Additional file 1: Table S2). The PO anatomy enrichment results of the seed and the total genes in all modules are respectively related to 26 and 20 different tissue/organs. For the tissue/organs covered the most genes in each module, 145 ones contain over 90% of the total genes in their current modules. This information could be helpful for choosing the right experimental conditions to study the co-expression relationship among genes of the same module.

To derive more detailed function for each of the 2,438 candidate genes, several software tools were utilized (Additional file 1: Table S5): 181 genes encode CAZy proteins [27]; 269 genes encode enzymes targeted to functions in Golgi, as predicted by GolgiP [28]; 161 genes encode transporters according to TransportDB [29]; and 657 genes encode proteins with at least one transmembrane domain, based on TMHMM [30]. In addition, a total of 144 TFs were found in 102 out of the 217 modules, belonging to 45 protein families (Table 1), most of which may be the main transcription regulators of the corresponding modules (Additional file 1: Table S6). Many of these transcription regulators have been experimentally verified to regulate the secondary cell-wall synthesis or the biomass formation [25,31-33], such as members of the MYB, NAC and WRKY families. 

**Table 1:** TFs belong to 45 protein families

**TF families**	**Numbers of genes included**	**Genes**
C2H2	12	AT1G10480,AT1G34370,AT1G68360,AT2G02070,AT3G02790,
		AT3G44750,AT3G62240,AT4G35700,AT5G10970,AT5G43170,
		AT5G54630,AT5G66730
bHLH	11	AT1G10120,AT1G71200,AT2G24260,AT3G07340,AT3G23690,
		AT3G26744,AT3G59060,AT4G36540,AT5G01310,AT5G48560,
		AT5G58010
HB	8	AT2G46680,AT3G01470,AT3G61890,AT4G02560,AT4G08150,
		AT4G34610,AT4G40060,AT5G02030
AP2-EREBP	7	AT1G28160,AT1G68840,AT2G44940,AT3G15210,AT3G16770,
		AT3G25890,AT4G39780
MADS	7	AT1G77950,AT1G77980,AT2G03710,AT2G24840,AT3G02310,
		AT5G10140,AT5G20240
NAC	7	AT1G01010,AT1G01720,AT1G52880,AT4G35580,AT5G04400,
		AT5G09330,AT5G13180
bZIP	6	AT1G08320,AT1G35490,AT3G54620,AT5G10030,AT5G15830,
		AT5G65210
HMG	6	AT2G17560,AT3G28730,AT3G51880,AT4G23800,AT4G35570,
		AT5G23420
AUX-IAA	5	AT2G33310,AT3G04730,AT3G23050,AT5G25890,AT5G65670
C3H	5	AT1G30460,AT2G02160,AT3G48440,AT3G51950,AT5G18550
MYB	5	AT1G18570,AT2G37630,AT2G38090,AT5G12870,AT5G14340
WRKY	5	AT1G29280,AT3G58710,AT4G01720,AT4G31800,AT5G07100
PHD	4	AT1G79350,AT2G36720,AT3G01460,AT3G51120
ARF	3	AT1G19220,AT1G19850,AT5G20730
C2C2-CO-like	3	AT1G68520,AT2G24790,AT5G24930
GARP-G2-like	3	AT1G25550,AT1G69580,AT4G18020
GRAS	3	AT1G55580,AT2G04890,AT3G54220
LIM	3	AT2G39900,AT2G45800,AT3G61230
SBP	3	AT3G57920,AT5G18830,AT5G43270
TCP	3	AT2G31070,AT4G18390,AT5G08070
TLP	3	AT1G47270,AT2G47900,AT3G06380
ZF-HD	3	AT1G74660,AT1G75240,AT4G24660
ABI3-VP1	2	AT2G30470,AT2G35310
CCAAT-HAP3	2	AT2G13570,AT2G38880
CPP	2	AT3G22760,AT4G14770
GRF	2	AT2G36400,AT4G37740
PcG	2	AT1G14030,AT5G42400
ZIM	2	AT1G17380,AT5G13220
ARID	1	AT1G04880
AS2	1	AT2G42430
BES1	1	AT5G45300
C2C2-Dof	1	AT5G39660
C2C2-YABBY	1	AT2G45190
CCAAT-HAP2	1	AT1G30500
CCAAT-HAP5	1	AT1G54830
EIL	1	AT5G21120
FHA	1	AT3G54350
HSF	1	AT4G36990
LUG	1	AT2G32700
MBF1	1	AT2G42680
TAZ	1	AT5G67480
Trihelix	1	AT1G76890
ULT	1	AT4G28190
VOZ	1	AT1G28520
Whirly	1	AT1G14410

### Identification and functional annotation of *cis* regulatory motifs

For each co-expression module, we have examined if genes in the module may be transcriptionally co-regulated by checking if the promoter regions of these genes share conserved sequence motifs as potential regulatory elements, using the CGMD pipeline (see Methods). Overall, 1,329 non-redundant motif instances were predicted (Additional file 1: Table S7), covering the promoters of 2,661 genes (Additional file 1: Table S8), representing 1,329 highly conserved motif groups (see Methods). 197 of the 217 modules (91%) each contain at least one conserved motif shared by at least 80% of the genes in the module (Additional file 1: Table S2 and Additional file 2: Figure S2a), providing a strong evidence that most genes in the same module are transcriptionally co-regulated. For the all motifs in each module, we also provided the p-values using BOBRO (Additional file 1: Table S2) [34].

Sequence comparison with known *cis* regulatory motifs in the PLACE and AGRIS databases reveal that 769 of the 1,329 motifs (58%) match well with 622 of the 1,009 annotated motifs (61%), indicating the high quality of our prediction (Additional file 1: Table S9). Out of the 1,329 motifs, 20 are palindromic (Additional file 1: Table S7). For the 201 novel genes with annotated PCW stage information, they share 273 conserved motifs with known PCW genes in the same stage (Additional file 1: Table S4). All these demonstrate the high quality of our predicted co-expression gene modules.

To assess the prediction specificity, we have checked a null hypothesis that the number of the known motifs in AGRIS and PLACE matched by our predicted motifs is essentially the same to the number of such motifs matched by predicted motifs based on groups of arbitrarily selected genes from the whole *Arabidopsis* genome using a Chi-square test (see Additional file 1: Table S10 for detail) [35]. The test rejected the null hypothesis with a p-value, 2.8e-05, indicating the high statistical significance of our predicted motifs.

We have also checked if the 1,329 predicted motifs are present in the promoter sequences of their corresponding orthologous genes in *Populus,* using the *fuzznuc* program of the EMBOSS package [36]. We identified 1,489 pairs of orthologous genes between *Arabidopsis* and *Populus* (Additional file 1: Table S5), covering 53% of the 2,787 genes (the 349 known and 2,438 candidate PCW genes). We scanned the promoter sequences of 1,489 genes of *Populus* using the 1,329 predicted motifs. Our search found that 374 of the 1,329 motifs (29%) were conserved in 1,234 out of the 1,489 pairs of orthologous genes (Additional file 1: Table S7), containing 149 known PCW genes and 1,085 candidate genes. We therefore conclude that these 374 motifs are more likely to be functional motifs and the corresponding 1,085 *Arabidopsis* genes may represent the most reliable prediction of the PCW genes.

### Location preference and abundance in the promoter regions of the predicted motifs

**Figure 3:**
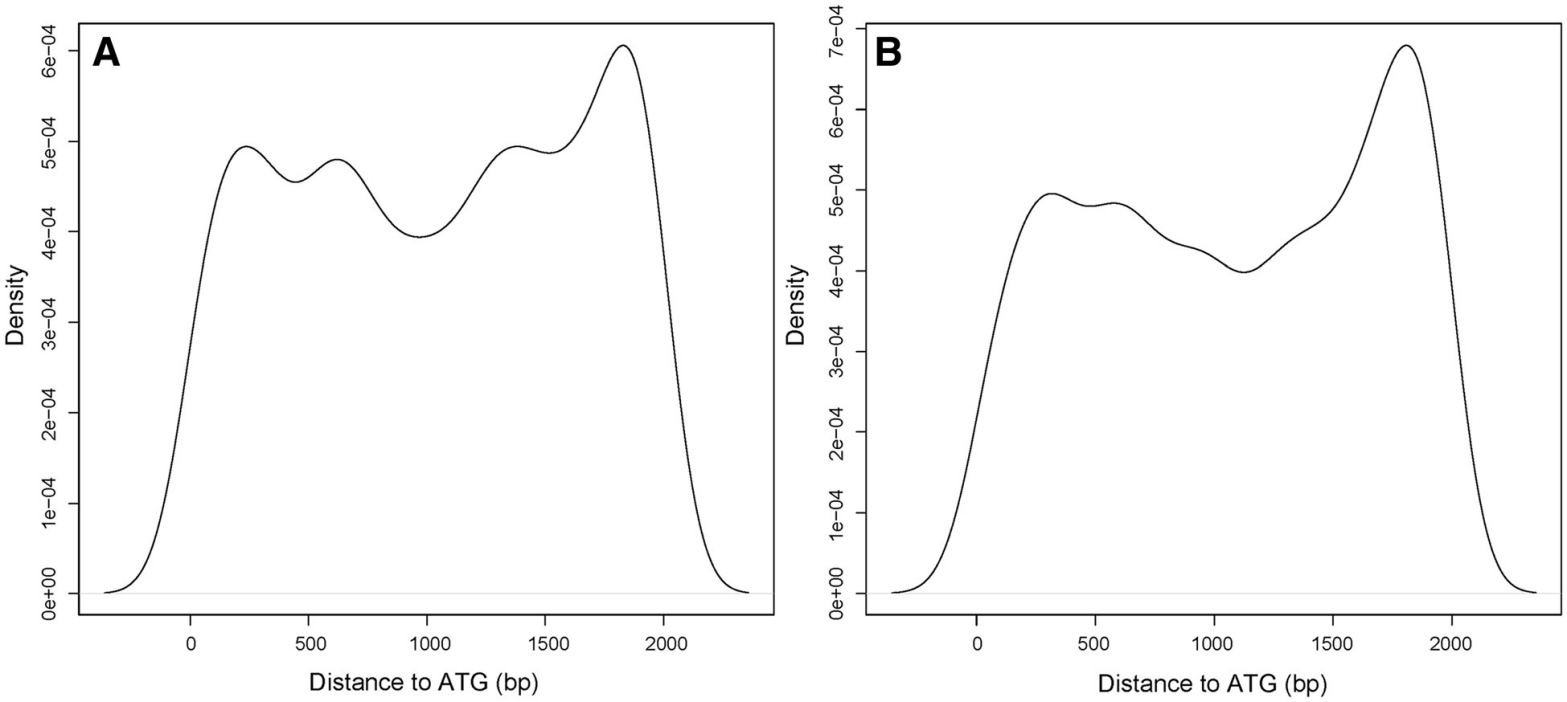
**Positional distribution of AC elements.** (**A**) Location distribution of the AC-I element-related motifs. (**B**) Location distribution of the AC-II element-related motifs.

Out of the 1,329 conserved motifs, 172 are predicted to be the binding sites of MYB related TFs, and 23 to be the binding sites of lignin biosynthesis related TFs (Additional file 1: Table S7). It is known that different *cis* regulatory motifs may have different preferences in terms of their locations in the promoters [37-40]. Here we use the AC element-related motifs as a case study, which are known to be present in the promoters of most lignin biosynthetic genes [32,41]. We found that 11 and 10 motifs in our identified motifs correspond to the AC-I and AC-II elements, respectively. For each of the two elements, we calculated the distance between the translation start site and the AC element of each lignin synthesis gene; and plotted the distribution of the distances. The two distributions are largely similar except that the AC-II element-related motifs have a higher percentage located between 1,750 bps and 2,000 bps away from ATG than the AC-I element-related motifs (Figure 3). This may suggest the potential difference between the two AC elements in terms of their locations in the promoter regions. 

**Figure 4:**
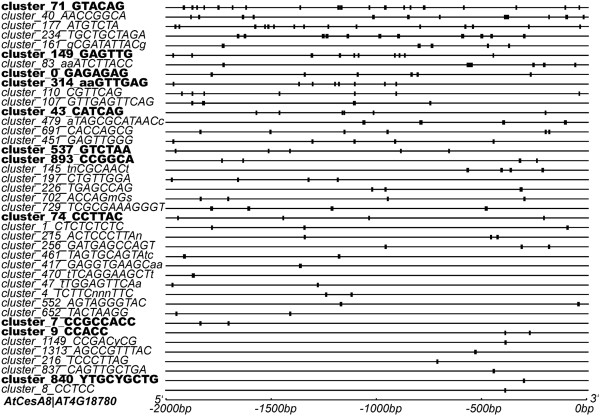
**Location distribution of predicted motifs in the promoter region of AtCesA8.** The IDs and patterns of motifs are shown on the left. Bold IDs indicate the motifs are conserved in the orthologous genes of *Populus*. Each black box on the right shows the occurrence of the motif. Motifs are ordered according to their copy numbers.

We also noted that one gene may have multiple unique motifs in its promoter region and one motif could appears multiple times (e.g. as very similar instances) in the same promoter. We noted that most of the annotated/predicted PCW genes each have at least five distinct motifs (Additional file 1: Table S8) and a motif sequence could have up to 50 copies in the promoter regions of the genes under consideration (Additional file 2: Figure S2b-c). The location distribution and abundance of motifs in one gene’s promoter region may play an important role in the regulation of gene expression [42-45]. For instance, Figure 4 shows that AtCesA8 (AT4G18780) has 41 unique motifs in its upstream region and each motif has one to 24 copies; 11 of them (bold font in Figure 4) are also found in the promoters of the CesA8 orthologous gene in *Populus*, hence indicating that such motifs are conserved during evolution. Specifically, motif *cluster_71_GTACAG* has the most number of copies and is conserved in both *Arabidopsis* and *Populus*. This motif matches the ABRE3 motif (GCCACGTACA) in PLACE, which is related to drought, low-temperature or high-salt stress (Additional file 1: Table S9). In addition, *cluster_9_CCACC* in the upstream of CesA8 is a variant of the AC element bound by MYB TFs [46]. 

### Functional prediction for co-expression modules

For each identified co-expression module, we have inferred the general functionality of the module as a whole based on the functional annotations of its known PCW genes. Specifically, we focused on modules containing four groups of seed genes related to PCW synthesis, namely cellulose syntheses, genes of the lignin synthesis pathway, genes of the nucleotide diphosphate sugar (NDP-sugar) synthesis, and genes of selected GT families (GT8, GT31, GT34, GT37,GT43, GT47) [47]. As a result, we identified 108 such modules: 30 modules are related to cellulose syntheses; 28 related to NDP-sugar interconversion; 38 related to lignin syntheses; and 49 related to GT families (Additional file 1: Table S11). In these 108 modules, 56 contain TFs, which might play a role in the regulation of the synthesis of some specific PCW components (Additional file 1: Table S2).

We also compared the consistency level between the functional annotations of each of the 108 modules and those of their corresponding predicted *cis* regulatory motifs for the module. Specifically, we first selected three most reliable motifs in each module (Additional file 1: Table S2) based on the number of genes covered and the conservation between *Arabidopsis* and *Populus*, and compared if the functional annotations of these motifs are consistent with the functional annotations of the module, i.e. check whether each motif belongs to one of the aforementioned MYB or lignin-synthesis related motifs (Additional file 1: Table S7). Of the 108 modules, 37 (34%) have consistent functional annotations from the two sources (Additional file 1: Table S2). For example, in a lignin-synthesis related module *86_1*, the most reliable motifs are *cluster_11cluster_56*, and *cluster_284*, which are respectively annotated to be known AC elements and two MYB binding sites, previously implicated in regulation of lignin biosynthesis [46,48,49]. In a cellulose-synthesis related module *119_1*, one of the three motifs, *cluster_149*, is annotated to be a binding site of the R2R3-type MYB TF, which is known to be involved in cellulose syntheses [50,51]. In addition, out of the 108 modules, 30 have un-annotated motifs. By excluding these, the overall consistency between the two annotated sources is 47%, indicating our functional inferences are generally reliable.

In the 108 modules, we identified the ‘hot links’ that are essential for PCW synthesis. The idea is that a few of high activity interactions might dominate the biochemical activity of the whole genetic network, comparing to the surrounding less active interactions [52]. Among the 108 modules, there are 119 groups of ‘hot links’ found in 68 modules; 52 such ‘hot links’ groups contain 98 seed genes of the four aforementioned groups (Additional file 1: Table S12) and 293 candidate genes. These ‘hot links’ represent the most dominated co-expression relationships in the 108 modules. For example, CESA4, CESA7 and CESA8 have been previously reported to form a protein complex for cellulose synthesis [12,17,23].

## Conclusion

Co-expression analysis has been widely used for identification of functional genes. In this study, we predicted new candidate genes related to PCW in *Arabidopsis* at a genomic scale. Compared to previous studies, this study has several novelties and advantages. First, we used a bi-clustering technique to analyze transcriptomic data collected multiple conditions, which represents an alternative method of traditional clustering for identification of co-expressed genes under some but not necessarily all provided conditions. Second, we used the co-expression relationships with all known PCW genes as seeds (rather than a few) to identify new candidate genes, which led to the identification of a significantly larger set of new candidates compared to previous studies. Third, we used a network topology-based approach to identify highly co-expressed gene modules within each network, which makes our prediction more reliable. Fourth, using a combination of three motif prediction tools, our motif prediction is more reliable, which is evidenced by our functional prediction consistency assessment. Lastly, our functional prediction at both individual gene level and the module level is informative and reliable through using three complementary analysis methods. The statistical validation for each analytical step ensures the overall quality of our computational analysis results. We anticipate our approach and data represented here will be useful for other researchers working on gene expression data analysis and PCW synthesis.

## Methods

### Data collection and processing

The normalized transcriptomic datasets for *Arabidopsis thaliana* were downloaded from AraGenNet [17], which contains genome-scale gene-expression data collected under 351 non-redundant conditions. The original datasets are Affymetrix ATH1 *Arabidopsis* microarray datasets (22,810 probe sets × 1,428 ATH1 microarrays) in TAIR (http://www.Arabidopsis.org). The probe sets in this dataset represent 21,031 *Arabidopsis* genes among which (a) 1,558 are annotated transcription factors by the DATF database (Database of *Arabidopsis* Transcription Factors) [53] and (b) 810 matched biunique known PCW genes according to the Purdue database [6] except for four GT family 43 genes. The genome sequences of *Arabidopsis* (version 9), *Populus* (version 2.0) and *Rice* (version 6.1) and associated annotations, including protein-encoding sequences and intergenic regions, were obtained from TAIR, Phytozome (http://www.phytozome.net/poplar) and RGAP (rice.plantbiology.msu.edu), respectively. The basic data processing was done using in-house Perl scripts; and statistical analyses were performed using the R package (http://www.r-project.org).

### Bi-clustering analysis of gene expression data

To identify genes that are co-expressed with known PCW genes, we adopted a two-step bi-clustering approach to analyze the aforementioned microarray dataset, which is represented as a 21,031 × 351 matrix, a required format by the QUBIC program [21]. The key algorithmic idea of the QUBIC program is based on the graph representation of a microarray dataset, converting the bi-clustering problem into a graph problem [21].

A seed-containing matrix (810 × 351) was extracted from this matrix, where 810 is the number of the known PCW genes, called *seeds*, and 351 is the number of experimental conditions. In first step, we run QUBIC on the seed-containing matrix to identify co-expression bi-clusters among the seed genes. In the second step, we run QUBIC on the large matrix (21,031 × 351) to grow the identified bi-clusters on the seed matrix, i.e. to recruit additional genes that are co-expressed with the seed bi-clusters under the same conditions.

Most microarray analysis programs take discretized data matrix to reduce the computation complexity. We have also discretized all the expression values into three levels, -1, 0, 1, representing down-, no- and up-regulation, respectively. QUBIC provides the flexibility in discretizing expression levels ranging from –K to + K, for any fixed positive integer K [21]. We found that K = 1 works well for our study. QUBIC uses a parameter *c* within [0, 1] as a threshold for controlling the consistency level of the expression patterns among the co-expressed genes within a bi-cluster. To find an appropriate *c* value, we performed a simulation study, which suggests that the *c* value between 0.7 and 0.98 should give the best performance result for our bi-clustering analysis; hence we have carried out a grid-based search for an optimal c value within this range using 0.05 as the increment. Specifically we have searched for a two-value (*c*_*1*_*c*_*2*_) combination that gives the best AUC (*area under curve*) value for the *receiver operating characteristic* (ROC) curve analysis [54,55] (See Additional file 1: Table S13, S14, and support information for details).

### Construction of co-expression networks and modules

Genes in a bi-cluster are co-expressed under a sub-set of the 351 experimental conditions. To assess the similarity level of a detected co-expression bi-cluster, we have examined the correlation between the expression patterns of each pair of genes in the same bi-cluster. Specifically, for each bi-cluster we calculated the Spearman’s correlation coefficient *rho* between the expression patterns of each pair of genes under the conditions associated with the bi-cluster. Note here we used the actual expression values instead of the discretized data (i.e. -1, 0 and 1). Gene pairs with *rho* > 0.7 (positive co-expression) or < −0.7 (negative co-expression) were considered as *significantly co-expressed*. This cutoff has been used by numerous published papers [11,56,57]. A bi-cluster was removed from further consideration if none of its gene pairs satisfy this cutoff.

For each bi-cluster passing this test, we constructed a *co-expression network* using Cytoscape [58] as follows: each node in the network represents a unique gene and each edge represents two genes with similar gene-expression patterns above the *rho* threshold under the conditions of the current bi-cluster. It should be noted that not all genes are equally co-expressed within a network; and each network generally consists of multiple clusters of highly co-expressed genes while inter-cluster co-expression relationships tend to be substantially weaker, hence having sparse edges. To identify all clusters of highly co-expressed genes within a network, we have applied a popular graph-based clustering algorithm "Molecular complex detection" (MCODE) [59], a plug-in of Cytoscape, to identify all (non-overlapping) clusters of highly co-expressed genes, each called a *co-expression module.* Specifically, each module is a connected sub-network with a substantially higher density of edges within the sub-network compared to the density between the sub-network and the rest of the network. The default scoring parameters in MCODE have been optimized to fit the average network well and hence we used them (see the manual of MCODE for details). Note that not all genes in a network are assigned to a co-expression module. It is the specified density level that determines which genes are selected or not. Actually we used this strategy to get rid of accidental predictions of co-expressed genes. When setting the density threshold, we intentionally set it high enough to rule out as many such accidental predictions as possible, which could also exclude some real co-expressed genes.

The final set of co-expression modules are derived from all the networks representing the bi-clusters identified above. Since some of the bi-clusters may have overlaps, i.e., some genes may be co-expressed with different sets of genes under different conditions. Hence the final set of co-expression modules may have overlaps. Such information allows us to infer the cellular-level functional relationship among co-expression modules containing overlapping genes.

### Prediction of conserved motifs

To determine if co-expressed genes in the same module are transcriptionally co-regulated, we have examined if they share conserved *cis* regulatory elements in their promoters. To this end, we have implemented a new pipeline, co-expression gene motif discovery (CGMD), to identify conserved sequence motifs in the promoter sequences of the relevant genes through integration of the prediction results by multiple algorithms, detailed as follows.

To acquire the promoter sequence of each gene in a co-expression module, we extracted an upstream region of 2,000 bps from the translation start site; we did not use the transcription start for this purpose since the current prediction of transcription start sites tends to be not very accurate. In addition, we used a 2,000 bps sequence as the core promoter because the length of a plant promoter is typically about 1,000 bps, plus the length of a 5’ un-translated region in *Arabidopsis* could be as long as 1,000 bps as our data showed (Additional file 2: Figure S3a).

For motif prediction, we used the following three prediction programs: WeederTFBS 1.4.2 [60], MotifSampler 3a [61,62] and PhyloCon 3.2 [63]. These programs were selected because of their recognized strong performance as well as the complementary nature among the programs [64]. WeederTFBS allows the motif length to be 6, 8, 10, or 12 bps long, and it outputs the 15 highest scoring motifs for each run; to-be-identified motifs were assumed to appear in all the underlying sequences; and each motif was allowed to appear more than once in a sequence. MotifSampler uses a prior probability in finding a motif, and sets the default length of the predicted motif at 8 bps. PhyloCon requires phylogenetic information for its motif prediction (the other two do not) so we need to provide orthologs of each concerned *Arabidopsis* gene in *Populus* and *Rice*, which we did using the bi-directional best hit approach [65] and predicted each motif that is conserved across the three orthologous sequences. For promoter sequences in the other two genomes, we extracted an upstream sequence of 2,000 bps for each *Populus* gene and an upstream sequence of 4,000 bps for each Rice gene from the translation start site of the gene. The reason is that for the Rice genome, a 5’ un-translated region could be as long as 3,000 bps while for *Populus,* its 5’ un-translated region is no more than 1,000 bps (Additional file 2: Figure S3b-c).

We have used CompariMotif [66] to integrate all the predicted motifs by the three programs, particularly highly similar predictions among the co-expression modules. Specifically, a similarity score for each pair of predicted motifs was calculated as the number of matched positions divided by the length of its maximum align-able positions between the two motifs. Based on this score, we then used MCL v10-201 [67] to cluster all the predicted motifs into groups, each of which has a similarity score above a predetermined threshold (the granularity parameter of MCL set at 4). We then aligned the motifs within each group (or cluster) using MAFFT v6.603b [68], and calculated a consensus sequence from the gapless multiple-sequence alignment of the motifs using the *cons* program of EMBOSS v6.2.0 [36] and used such consensus sequence as the representative of each motif group.

To annotate the function of such motifs, we have compared the resulting motifs from the above analysis with the known motifs in the two plant motif databases: AGRIS [69] and PLACE [70] by using CompariMotif. For motifs in the two databases, we also performed an integration of the best representative from each cluster as done above. For each pair of compared motifs, if their similarity score is > 4 and the percentage of their matched positions >80%, they were considered as essentially the same motif.

To assess the statistical significance of a predicted consensus motif, we have compared the numbers of the known motifs in AGRIS and PLACE matched by the predicted motifs using two different methods, which are separately based on co-expression genes and groups of arbitrarily selected genes from the whole genome of *Arabidopsis*. Specifically, we created 1,000 arbitrary gene groups with the same size as the average size of all the co-expression modules under consideration. For each such gene group, we predicted motifs using the above procedure (WeederTFBS only). To be consistent, we did motif prediction for the co-expressed genes using WeederTFBS only for this comparison purpose. Our null hypothesis is that the proportion of the known motifs matching the predicted motifs among the co-expressed genes is the same for that of the arbitrarily selected genes. A Chi-square test was employed to test this hypothesis [71]. Based on the Chi-square test p-value on the given datasets, the hypothesis can be rejected or accepted.

## Abbreviations

Purdue database: Purdue Cell Wall Gene Families database; CESA: Cellulose synthesis; known PCW genes: Known/annotated PCW related genes; TFs: Transcription factors; PO: Plant Ontology; NDP-sugar: Nucleotide diphosphate sugar; PCW: Plant cell-wall; DATF database: Database of *Arabidopsis* Transcription Factors; AUC: *Area under curve*; ROC: *Receiver operating characteristic*; CGMD: Co-expression gene motif discovery; GT: Glycosyl transferase; GATL1: Galacturonosyl transferase-like 1; Parvus: Polygalacturonate 4-alpha-galacturonosyltransferase; GAUT: Galacturonosyl transferase; IRX: Irregular xylem; FRA: Fragile fiber; ABRE: Abscisic acid response element; MYB: Myeloblastosis viral oncogene homolog; AC element: Activator element.

## Competing interests

The authors declare no competing interests.

## Authors’ contributions

SW planned and conducted the whole project, analyzed data, and wrote the manuscript. YY initiated and supervised the project, analyzed data, and revised the manuscript. QM planned the bi-clustering analysis, and provided technical supporting for using QUBIC and BOBRO program. XT provided technical supporting for the discretization of the microarray data matrix. DH provided support and guidance. YX provided support and guidance, initiated the project, analyzed data, and revised the manuscript. All authors read and approved the final manuscript.

## Supplementary Material

Additional file 1 **Supplementary Tables.** Supplementary Tables S1-14.) Click here for file

Additional file 2 **Supporting information.** The details for ROC curve analysis and supplementary Figures S1, S2 and S3. Click here for file
