# Supplementary material for: Genome-scale identification of cell-wall related genes in Arabidopsis based on co-expression network analysis
Source: BMC Plant Biol. 2012 Aug 9;12:138. doi: 10.1186/1471-2229-12-138 (PMC3463447; doi:10.1186/1471-2229-12-138)
Supplement: Additional file 1 — Supplementary Tables. Supplementary Tables S1-14.) [file 1471-2229-12-138-S1.doc]

**Supporting information**

According to the GO annotation and the Purdue database, we have chosen 12 GO terms unrelated to PCW synthesis (Table S13), and selected 861 genes that are assigned with the 12 GO terms as the negative dataset. For each *c* value pair, we randomly selected five sixth of the 810 seed genes as the training data and the remaining one sixth as the positive dataset; and do this for 1,000 times. The AUC (area under curve) of the ROC (receiver operating characteristic) curve was calculated based on the 1,000 time simulation. We found that the AUC has the highest value when *c_1_* = 0.85 and *c_2_* = 0.90 (Table S14).

**Calculation of the area under the ROC curve:**

*AUC = (TPR1 + TPR2) × (FPR2- FPR1) ×0.5+ (TPR2 +0) × (TPR2-0) ×0.5*

*(Y axis) TPR = TP / (TP+FN) = found seed genes/135*

*(X axis) FPR = FP/ (FP+TN) = found false set genes/861*

**If the value of FPR is equal for two distinct values of TPR, we use an average value of FPR for the two distinct values of TPR.*

*TP: true positive; FP: false positive; TN: true negative; FN: false negative; TPR: true positive rate;* and *FPR: false positive rate;*

**Figure S1**: **Size (the number of genes) distribution of the 217 co-expression modules.**


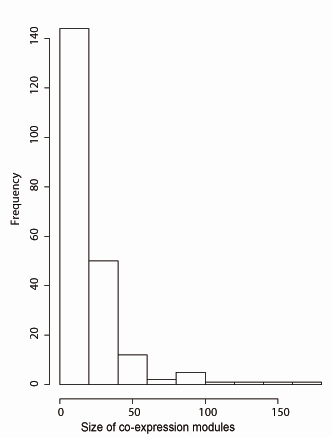


**Figure S2**: **Statistics of the 1,329 unique motifs.**

**Figure S3**: **Length distributions of 5’ UTRs in the three plant genomes.**
